# Automatic detection and measurement of viral replication compartments by ellipse adjustment

**DOI:** 10.1038/srep36505

**Published:** 2016-11-07

**Authors:** Yasel Garcés, Adán Guerrero, Paloma Hidalgo, Raul Eduardo López, Christopher D. Wood, Ramón A. Gonzalez, Juan Manuel Rendón-Mancha

**Affiliations:** 1 Centro de Investigación en Ciencias, Instituto de Investigación en Ciencias Básicas y Aplicadas, Universidad Autónoma del Estado de Morelos (UAEM), Cuernavaca, Morelos, México; 2 Centro de Investigación en Dinámica Celular, Instituto de Investigación en Ciencias Básicas y Aplicadas, Universidad Autónoma del Estado de Morelos (UAEM), Cuernavaca, Morelos, México; 3 Instituto de Biotecnología, Universidad Nacional Autónoma de México (UNAM), Cuernavaca, Morelos, México; 4 Laboratorio Nacional de Microscopía Avanzada, Instituto de Biotecnología, Universidad Nacional Autónoma de México (UNAM), Cuernavaca, Morelos, México

## Abstract

Viruses employ a variety of strategies to hijack cellular activities through the orchestrated recruitment of macromolecules to specific virus-induced cellular micro-environments. Adenoviruses (Ad) and other DNA viruses induce extensive reorganization of the cell nucleus and formation of nuclear Replication Compartments (RCs), where the viral genome is replicated and expressed. In this work an automatic algorithm designed for detection and segmentation of RCs using ellipses is presented. Unlike algorithms available in the literature, this approach is deterministic, automatic, and can adjust multiple RCs using ellipses. The proposed algorithm is non iterative, computationally efficient and is invariant to affine transformations. The method was validated over both synthetic images and more than 400 real images of Ad-infected cells at various timepoints of the viral replication cycle obtaining relevant information about the biogenesis of adenoviral RCs. As proof of concept the algorithm was then used to quantitatively compare RCs in cells infected with the adenovirus wild type or an adenovirus mutant that is null for expression of a viral protein that is known to affect activities associated with RCs that result in deficient viral progeny production.

Virus replication can induce extensive rearrangement of cellular components that results in de novo formation of specialized intracellular compartments where the viral genome is replicated. Depending on the virus family such compartments, which have been termed viroplasms, virus factories, replication centers or compartments (RCs), may associate with cellular membranes, the cytoskeleton, or nuclear domains^1^,^2^. In every case RCs assemble complex macromolecular platforms, where viral and cellular proteins responsible for viral genome replication are concentrated, thus increasing viral replication efficiency. Interestingly, the same compartments recruit, regulate and co-opt cellular factors that participate in a variety of host defense mechanisms. Therefore, RCs seem to act as molecular hubs where many aspects of virus-host cell interaction are controlled and there is considerable interest in understanding the impact of their formation on virus replication as well as on the cellular activities that are altered as a consequence of their assembly.

Like other DNA viruses that replicate in the cell nucleus, adenoviruses induce formation of RCs (AdRC) that assemble in association or adjacent to specific nuclear domains (ND). However, most aspects of the structure and function of AdRC remain to be explored; some of these include the relationship between their molecular components and the activities they regulate, as well as the molecules involved in their structural integrity and the dynamics of their assembly.

A strategy that has been commonly used to study RCs has relied on the phenotypic analysis of recombinant viruses that harbor substitutions or deletions in the genes that are known or suspected to affect RCs formation or activities. Such studies have typically used fluorescence microscopy to screen for alterations in the morphology or dynamics of formation of RCs at different times of the viral replication cycle^3^. Because the viral DNA binding protein (DBP) associates with ssDNA, it is a useful marker to study RCs by fluorescence microscopy. Analysis by fluorescence microscopy of AdRC formation through the viral replication cycle has shown that the DBP protein initially accumulates in small foci that seem to increase in size and number resulting in doughnut-shaped, crescent or spherical structures. Very late during the replication cycle, the latter structures appear to coalesce resulting in complex amorphous structures that may occupy most of the cell-nucleus. Although informative, these studies have major drawbacks and provide limited information because they are qualitative and depend on subjective visual analysis for classification of the RCs at different stages of assembly. Previous data on the visual analysis of RCs suggest that these structures can be approximated as ellipse-shaped structures, a potentially oversimplified approach considering the complexity and variability of their number, shape and size during the viral replication cycle (see Fig. 1). Nevertheless, the use of ellipses allows for considerable data reduction and simplification, and consequently provides a fast and simple image analysis pipeline to be employed.

Some of the best known image processing approaches for detection and adjustment of ellipses are the Hough Transform^4^,^5^,^6^, Random Sample Consensus (RANSAC)^7^,^8^,^9^,^10^ and least squares^11^,^12^,^13^,^14^. Grouping approaches (Hough Transform and RANSAC) are robust against noise and detect multiple ellipses, but are relatively slow, require a lot of memory and suffer from low accuracy. On the other hand, least squares approaches are comparatively fast and accurate, yet can only adjust one ellipse at a time and are sensitive to noise. In 1996 Fitzgibbon^15^ proposed a new algorithm for adjustment of ellipses in scattered data. The main idea is to force the least squares problem in a way that the solution is always an ellipse, taking into account the constraint 4*ac* − *b*^2^ = 1. The optimization problem is solved using Lagrange multipliers and generalized eigenvectors. Two years after the initial proposal of Fitzgibbon’s algorithm, Halir and Flusser presented a numerically stable version, incorporating a block decomposition strategy^16^. This is the most commonly used alternative for the implementation of the “Direct Least Square Fitting Ellipse” or DLSFE algorithm. This method is known to be a robust deterministic algorithm, invariant under affine transformations, computationally efficient and a non-iterative approach whose result is always an ellipse. A simple Matlab implementation is available in the paper of Halir and Flusser^16^. The DLSFE can only adjust one ellipse to a data set (it is not possible to adjust multiple ellipses) and it is very sensitive to outliers. For these reasons, the direct application of this algorithm in problems of image segmentation is not generally considered viable. To our knowledge RCs measurements have never been performed using ellipse adjustment, nor are there any reports where RCs have been analyzed making quantitative measurements.

In this work a very simple and efficient approach to detect RCs by adjusting ellipses is proposed. This new algorithm is automatic, deterministic, non iterative, can simultaneously detect multiple viral RCs and has a linear computational complexity. The algorithm was validated using a synthetic ground truth image base, and was then applied to real images obtained by fluorescence microscopy of Ad-infected cells processed at various time-points post-infection. As a proof of concept, it was then used to adjust and measure various parameters of AdRC in cells infected with the wild type virus (Ad wt) or with a virus mutant that does not express an adenoviral oncogene, which encodes the multifunctional protein E1B-55K, that is known to associate with RCs and affect various activities that result in inhibition of cellular defense mechanisms and production of viral progeny. Using a set of useful parameters: the number of RCs per cell nucleus; the area of RCs within nuclei; the intensity of the signal associated with RCs; and the ellipse eccentricity, as a measure that facilitates distinction between different RCs morphologies, the method allowed the automatic determination of a previously unrecognised effect of this mutation on the dynamics of formation of AdRC.

## Results

### Segmentation of the viral replication compartments

**Figure 1:**
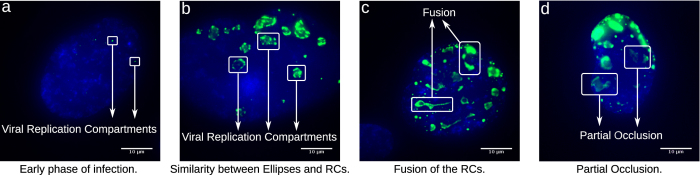
Examples of Ad-infected cells, at various times post-infection. The green color marks the viral replication compartments revealed by DBP staining, and DNA is marked with DAPI staining in blue. (**a**) AdRC appear as foci during the early phase of the replication cycle. (**b**) During the late phase AdRC form doughnut, crescent or spherical structures. (**c**) Very late in the replication cycle some RCs coalesce resulting in amorphous structures. (**d**) In the viral replication cycle, some RCs can present partial occlusion in some areas. For these four images the contrast of the images was increased to improve the visualization.

To study the dynamics of the formation of AdRC, we developed a new algorithm that provides an efficient approach to automatically detect and measure RCs by adjusting ellipses. Fluorescence microscopy images generated as described in the methods section were used. The fluorescent staining of DBP was used as a bona fide marker to detect RCs, which were approximated through ellipses at various times of the viral replication cycle. As shown in Fig. 1, very late in the replication cycle, RCs may coalesce forming complex amorphous structures. The following steps summarize the work-flow of the algorithm (described in detail in the methods section):

1. Filter RCs zones: This step consists in thresholding the image in order to segment the zones of interest(remove noise and atypical values). Our proposal performs an automatic selection of threshold value for each image **I** (*n* × *m*) considering the mean value (*M*_**I**_) and the standard deviation (*S*_**I**_) per column of the image. The threshold value is defined by:


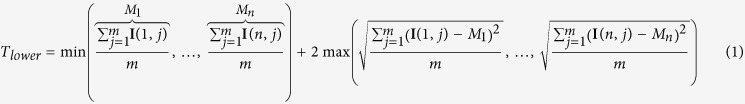



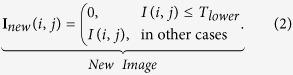


2. Compute the connected components: The connected components labelling is used to detect connected regions in the image (there exists a digital continuous path between all pairs of points in the same component). This heuristic consists of visiting each pixel of the image and creating exterior boundaries using pixel neighbours according to a specific type of connectivity. These arcs belong to the viral replication centers because the algorithm only uses the information of the color that marks the RCs. The results of this step are *N* connected components *A*_*i*_, *i* = 1, 2, …, *N*, where each *A*_*i*_ is part of one RCs.

3. Adjust the exterior boundaries using ellipses: For each connected component *A*_*i*_ apply the DLSFE algorithm. For more details see the methods section.

**Figure 2:**
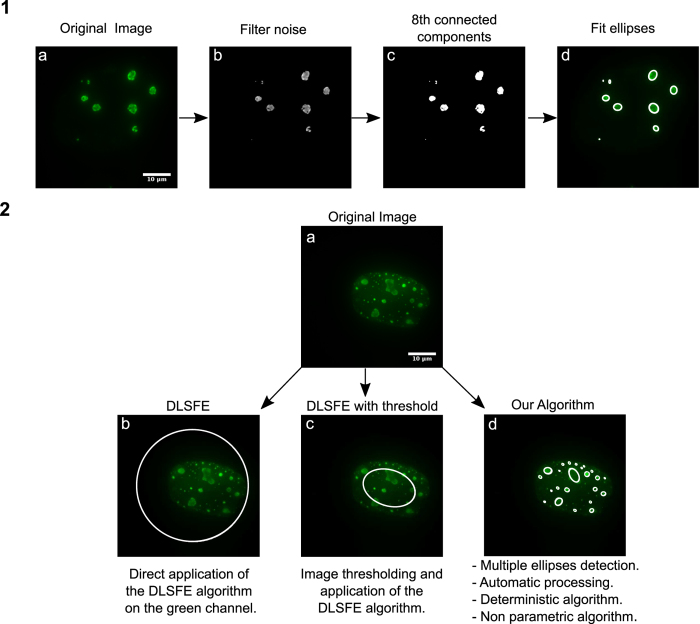
Visual comparison of our algorithm with other applications of the DLSFE algorithm. Panels 1a–d: Shows the steps of the proposed algorithm in a real image. Panel 2a: Original image. Panel 2b: DLSFE algorithm using all the pixels with non-zero value in green channel. Panel 2c: DLSFE algorithm application to the filtered image considering the threshold defined by Eq. (1). Panel 2d: Result using our algorithm.

Step 1 of the algorithm filters out noise and atypical values, step 2 divides the images in *N* connected components on the location of the RCs, and finally, the DLSFE adjusts each RC with an ellipse. These three steps constitute the adaptation of the DLSFE for detection and adjustment of multiple ellipses in images of any size or resolution. In the Fig. 2(1) we show graphically the steps of the proposed algorithm.

The results shown in panels 2b and 2c of Fig. 2 are consequences of eliminating some steps of the proposed algorithm. Both images show poor adjustment to RCs, because there is no delimitation of each RC. This is a consequence of the global adjustment by one ellipse. The results of our algorithm are shown in Fig. 2 panel 2d, where a good fit of the RCs is observed, suggesting this algorithm provides a tool that can be used to make automatic and quantitative measurements of RCs.

The algorithm presented in this work is automatic, deterministic, non-parametric, non iterative, and these results demonstrate that it can detect and adjust multiple ellipses of any size, eccentricity, position or rotation angle. Furthermore, this new method has a linear computational complexity and is invariant under affine transformations.

### Validation of the algorithm

The algorithm validation was performed using synthetic ground truth images generated taking into account diffraction, white noise, dark-current noise and signal amplification^17^. These images contain ellipses of different sizes, eccentricities, positions and rotation angles, for which real parameters of their implicit equations are known. This facilitates analysis of the algorithm’s response against several plausible events, hence permitting comparison of the obtained results with “ground-truth” ellipses considering different signal/noise ratios (SNR).

**Figure 3:**
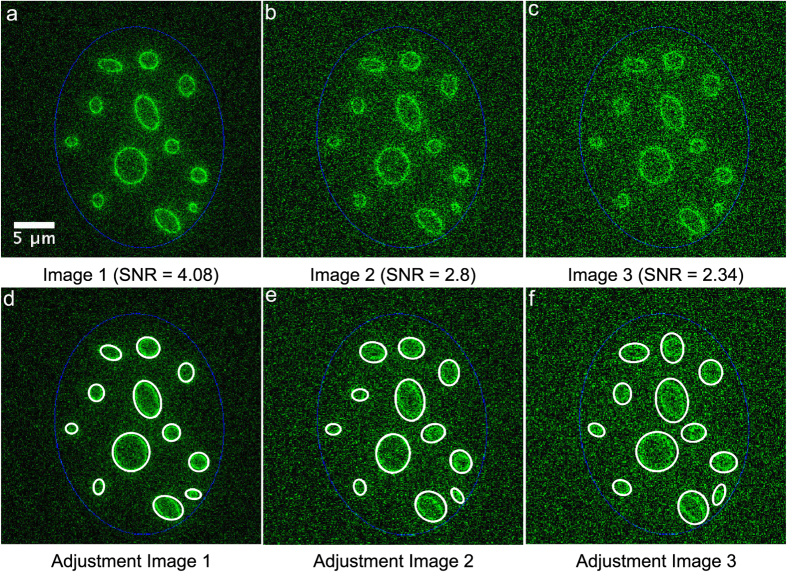
Adjustment of artificial images with different levels of Signal/Noise. The panels 3(a), 3(b), 3(c) show the synthetic images generated artificially. In the second row panels 3(d) to 3(f) show the results of our algorithm for these images. The blue ellipses simulate the nucleus of the cell.

The results are shown in Fig. 3, where panels 3(a) to 3(c) show some examples of these images considering three levels of noise and panels 3(d) to 3(f), the results obtained from these images. In each case we obtained an adequate adjustment regardless of the level of noise, size, position or eccentricity of RCs, demonstrating that this approach can be used to adjust multiple viral RCs through ellipses with a small error.

**Figure 4:**
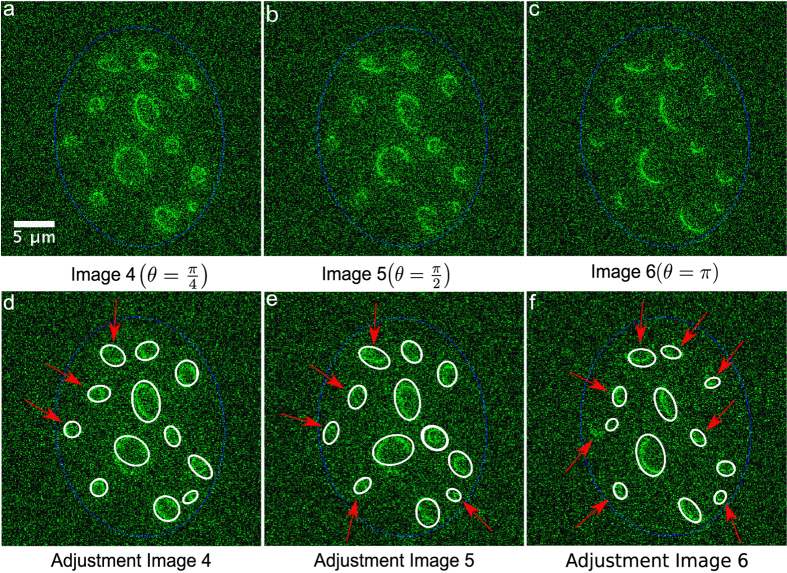
Adjustment of artificial images with partial occlusion (*SNR* = 2.34). The panels 4(a), 4(b), 4(c) show the synthetic images generated artificially with partial occlusion. In the second row, panels d to f, show the results of the algorithm for these images.

Microscopy images of viral RCs may present partial overlapping in some zones, which might lead to erroneous results for most segmentation algorithms (Fig. 1(d))^18^. This problem is significant when the aim is to obtain precise and quantitative measurements of the properties of RCs. Therefore, we tested the algorithm against images presenting partially overlapping RCs. For this, the algorithm was applied to twenty-five synthetic images presenting different levels of partial occlusion, and different levels of lack of information. Fig. 4(a–c) show three of these images considering different angles of partial occlusion.

Figure 4(d–f) show the algorithm outcome obtained for the synthetic images of 4(a), 4(b), and 4(c) respectively. In the test images all viral RCs were detected successfully. Regarding an approximate qualitative assessment of the accuracy of the adjustment, each partial occlusion angle determines the degree of information eliminated in the simulated viral RCs. In Fig. 4(d,e) an accurate adjustment of RCs is observed, even in zones where information is poor and can be confused with the image background (see the red arrows). In the case of *θ* = *π*, Fig. 4(f) shows results of decreased accuracy despite the detection of all RCs. In this example, where only 50% of the data for each image generated is preserved and can be considered as an extreme and unusual case (note that it is difficult to detect the position and form of some RCs by visual inspection), the algorithm can still adjust RCs, although with lower accuracy. The proposed method for detection and approximation of the RCs was evaluated quantitatively using synthetic images. Images were generated drawing ellipses from analytical formulas. The parameters (*a*, *b*, *cx*, *cy*) of analytical formulas are the ground truth used for evaluation. The rotation angle was not taken into account in this manuscript. However in Supplementary Information the performance of the proposed algorithm is scrutinized against the impact of noise over the identification of rotation angles.

In order to evaluate the thresholding process (Step 1) of the algorithm, we compared our proposed algorithm with four well known thresholding alternatives: Intermodes, MaxEntropy, Otsu and Yen. Intermodes smoothes the histogram in an iterative way until only two peaks remain^19^. MaxEntropy choses the threshold which maximizes entropies of distributions above and below the threshold^20^. Otsu’s method selects the thresholding that maximizes interclass variance and minimizes intraclass variance^21^. Yen’s method uses a maximum correlation criterion to select threshold^22^. The experiment was conducted taking into account that each image (one for each level of SNR) contains a set of synthetic RCs with different sizes, positions and eccentricities. For the validation we used the index of Jaccard, the Precision and the Recall in order to evaluate the performance of the methods in different scenarios like noise and partial occlusion. More details about these indexes and their application in our specific problem are explained in the methods section.

**Figure 5:**
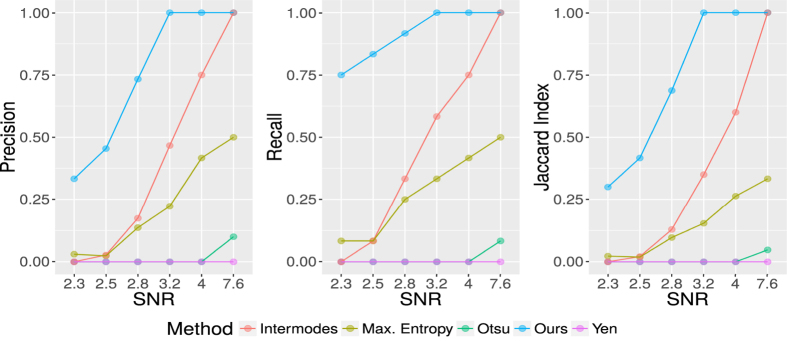
Precision, Recall and Jaccard indices for the detection and approximation of the viral RCs considering different levels of signal/noise in the synthetic images. Five different thresholding methods were tested (Intermodes, Maximum Entropy, Otsu, Yen and our method), each panel illustrates the results for these alternatives.

Figure 5 shows the obtained results for the Precision, Recall and Jaccard Index for 6 different Signal/Noise ratios (*SNR* = {2.3, 2.5, 2.8, 3.2, 4, 7.6}). With methods like Otsu, MaxEntropy and Yen we obtain bad results for all Signal/Noise ratios, even with high quality in the images (high Signal/Noise level). On the other hand, the best results can be observed when we use our method or the Intermodes alternative, in this case the Precision, Recall and Jaccard index are all superior with our proposal for any SNR. For these experiments we generated synthetic images with low Signal/Noise ratios (*SNR* = {2.3, 2.5, 2.8}), even though real microscopy images do not have such poor quality. With these SNR values the algorithm could detect succesfully some RCs (Precision, Recall, and Jaccard indices >0.25 in all cases). For images with SNR that are closer to expected values (*SNR* = {3.2, 4}), our method can correctly detect all RCS (Precision, Recall, and Jaccard indices equal to 1).

**Figure 6:**
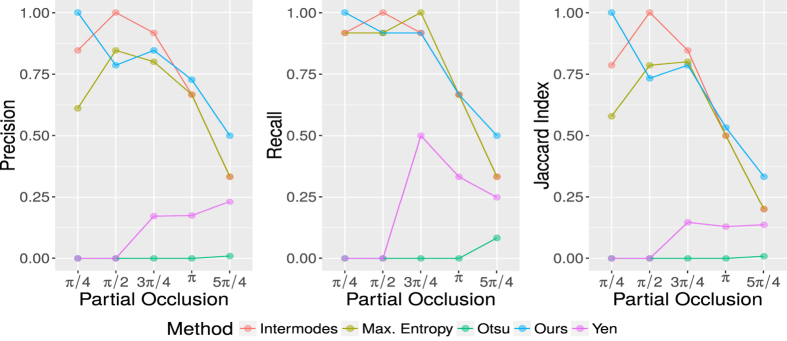
Precision, Recall and Jaccard indices for the detection and approximation of the viral RCs considering different levels of partial occlusion in the synthetic images. Five different thresholding methods were tested (Intermodes, Maximum Entropy, Otsu, Yen and our method).

Figure 6 shows the performance of our method compared with four alternative thresholding techniques in synthetic images with partial occlusion. For this experiment we consider a fixed SNR equal to 3.2 and five angles for partial occlusion *θ* = {*π*/4, *π*/2, 3*π*/4, *π*, 5*π*/4}. The SNR value of 3.2 was chosen as representative of the SNR “normally” found in epifluorescence images. The worst results are obtained with the threshold of Otsu and Yen. In these cases the Precision and Jaccard indices are always less than 0.25, while the Recall have a maximum value of 0.5 with a partial occlusion *θ* = 3*π*/4. The performance of the proposed algorithm by means of considering Intermodes thresholding is superior to the other tested thresholding algorithms for *π*/2 and 3*π*/4 degrees of partial occlusion. In these particular cases, the heights of the two peaks of the histogram computed from their corresponding synthetic images were comparably similar, therefore Intermodes outperforms other thresholding methods. However, such particular cases are far from being representative. In general, the results shown in Fig. 5 indicate that Intermodes thresholding does not guarantee the identification of the best threshold level for all images. Whatever the case, the data presented in Fig. 6 represent a particular adaptation of the proposed algorithm, which itself is completely compatible with any desired thresholding method. More thresholding algorithms might provide an advantage for other given particular cases, e.g. local thresholding will perform much better that global thresholding in images taken with heterogeneous illumination. When partial occlusion angles are within interval [0, *π*) the Precision, Recall and Jaccard indices are relatively high, i.e. the proposed algorithm is reliable when more than half of the data of interest are present.

### Analysis of viral replication compartments in Ad-infected cells

The algorithm was then used to analyze RCs in Ad-infected cells. Images of Ad-infected cells obtained at various times post-infection by fluorescence microscopy were grouped in eight different sets (12; 16; 20; 24; 28; 32; and 36 hpi). For each time-point, 60 images of infected cells were recorded, hence the results of this section represent the analysis of 420 images of the adenovirus infection process (a typical personal computer requires about 0.01 seconds to process a single image).

**Figure 7:**
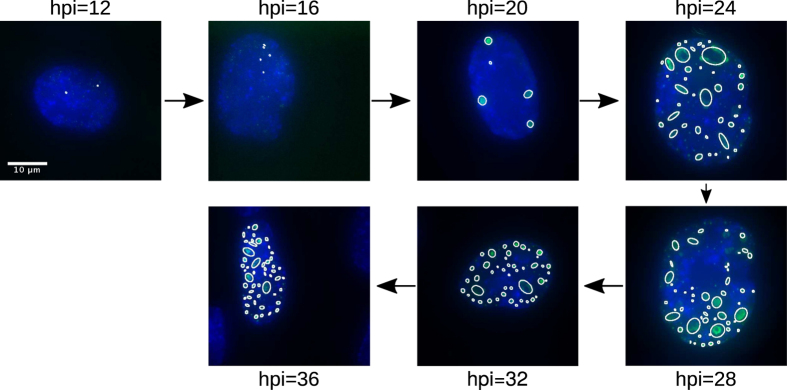
Detection and adjustment of the RCs in real images. A set of images each representative of a different time point of the viral replication cycle, and the results obtained using the algorithm are shown.

Figure 7 depicts representative results obtained for each time-point. At the earlier time-points (*hpi* = {12, 16}), before the onset of viral DNA replication, RCs are just becoming apparent, and those which were detected by the algorithm were small dot-like foci. At 20 hpi, although still scarce and isolated, RCs were larger and better defined. Qualitatively, in the time period between 20 and 32 hpi a significant rise in the number of RCs, as well as of their size and the fluorescence intensity, was observed. The results obtained at 36 hpi, showed an appreciable rise in the number of RCs in comparison with the previous time-points. At the latter time-points the larger RCs seem to coalesce, occupying a large proportion of the cell nucleus and forming complex amorphous structures, for which the adjustment of ellipses is no longer accurate (see Discussion section).

One of the main advantages of approximation through ellipses is the possibility to obtain quantitative data, e.g. position, area, perimeter, form and average fluorescence intensity of the RCs. These indicators allow for a statistic study of the behaviour of RCs at different time-points, providing relevant information about the viral replication cycle.

**Figure 8:**
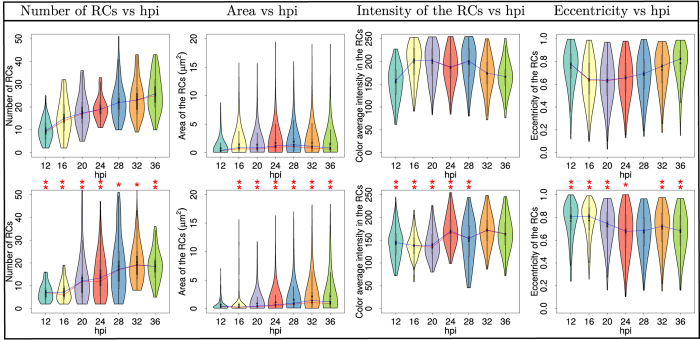
Descriptive statistical analysis of the number, area, mean intensity and eccentricity of the RCs. First row: Ad5 wt virus violin graphs (H5pg4100); second row: Ad5 mutant virus (H5pm4149). The number of cells analysed for each experimental condition was *n* ≥ 60. The blue line represents the mean and the red line the median. The red asterisks represent the statistical significance difference between the wild type and mutant virus using the test of Kruskal-Wallis^23^: 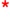
ρ-value ≤ 0.05; 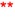
ρ-value ≤ 0.01.

Therefore, we conducted statistical analysis of the data obtained by the algorithm from processing images of cells infected either with the wt virus (Ad5 H5pg4100) or with a mutant virus (Ad5 H5pm4149), as described in the methods section. This virus mutant (E1B-) does not direct the synthesis of the early protein, E1B-55K, and has been shown to display defects in viral late gene expression, as well as in inhibition of cellular anti-viral defenses, resulting in decreased production of viral progeny. The results obtained are shown in Fig. 8, where violin plots of the number of RCs per cell nucleus; the area of RCs; their fluorescence intensity; and eccentricity of RCs were plotted against various time-points post-infection. For clarity, the analysis was divided into three different time intervals (*hpi* = {12–16}; *hpi* = {20−28}; *hpi* = {32–36}). The analysis of the data showed that except for few time-points and parameters (the area at 12 hpi; the intensity at 32 and 36 hpi; and the eccentricity at 28 hpi) there exists in all variables a statistically significant difference between the wild type and mutant virus according to the test of Kruskal-Wallis^23^.

At the time interval between 12 and 16 hpi, the violin plots (see Fig. 8) show that for the Ad5 wt virus there was a clear increase in the number of RCs, and a slight increase in their area and intensity. Interestingly, for the E1B- virus, while a slight rise in the number of RCs was observed, their area, intensity and eccentricity decreased in this time interval. Significantly, these data indicate that during the early times post-infection the algorithm is able to distinguish small changes in the number, size and shape of RCs. During the next time interval (20 to 28 hpi), viral genome replication initiates and the expression of viral late genes is known to increase several fold. In the Ad5 wt-infected cells, the number of RCs displayed further increase from the 16 hpi time point, and they reached their maximum area and intensity between 24 and 28 hpi. In contrast, cells infected with the E1B- virus displayed a complex behaviour, as the RCs increased in number between 16 and 20, but dropped slightly at 24 hpi. Interestingly, the area of RCs increased only transiently (at 24 hpi) before 32 hpi. The interval between 32 and 36 hpi corresponds to a phase during viral replication where viral late proteins and genomes are produced in large quantities and assembly of viral progeny reaches its highest level. The number of RCs detected in the Ad5 wt infected cells showed a steady rise during this interval; however, in contrast to the wt virus, where RCs reach a size that is nearly constant between the 20 hpi and 32 hpi time-point, the mutant virus showed again a complex behaviour in which RCs reached a maximum value that decreased not only in size but also in number after 32 hpi. An altered behaviour was also matched by the fluctuations in intensity and eccentricity of the RCs for the E1B- virus. For the Ad5 wt the maximum level of intensity after the initial rise at 16 hpi was reached at 28 hpi; in contrast a nearly opposite behaviour was observed for the E1B- virus as the initial rise that was observed only after 20 hpi was followed by a clear drop at 28 hpi. Interestingly, while the lowest values of eccentricity are reached in the 16–20 hpi interval in Ad5 wt, for the E1B- virus the lowest values are only reached at 24 hpi.

**Figure 9:**
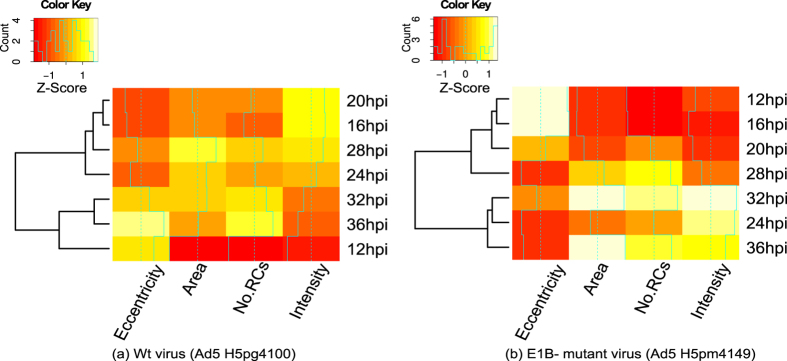
Hierarchically clustered heatmap for eccentricity, area, number of RCs and intensity, considering all times post-infection.

The hierarchical clustering maps presented in Fig. 9 for the Ad5 wt and E1B- mutant virus show some of the characteristics of RCs described above. The dendrograms indicate conglomerates according to qualitative and quantitative results: For the Ad5 wt virus the first group level contains classes {16, 20}, {32, 36}. The second group level contains {12, 32, 36}, {16, 20, 28}, while the third level contains {16, 20, 24, 28}. The latter group was an expected result because the items of each class are different to each other by 4 hours, and therefore, the results are more similar for any two time-points that are chronologically close to each other; however, it is interesting to note that such hierarchical clustering would be anticipated for the progressive formation of RCs as the viral replication cycle advances. In contrast, the results for the E1B- mutant virus display different sets of conglomerates, where the 12, 16 and 28 hpi time-points remain ungrouped in the first two or three levels, respectively, and seem to display an arrest, as there is no clear progression from the 12 hpi to the 20 hpi in the area, intensity or number of RCs, and a similar defect is apparent between the 24 and 28 hpi time points. These results indicate a defective progression from these two time-points, which correspond to the initial formation of RCs (12 hpi) and to the transition to the late phase of the viral replication cycle (20 to 24 hpi). The dendrograms also show that for both Ad5 wt and the E1B- mutant virus conglomerates tend to group around the 24, 28 and 32 time-points, suggesting these two time points may be of particular relevance for RCs during latter periods post-infection.

Significant differences between Ad5 wt and E1B- through the viral replication cycle were clearly detected by the algorithm, as in contrast to Ad5 wt, where the transition to the late phase of infection clearly showed an increase in the area and intensity of the RCs, as well as a shift of decreased eccentricity, the E1B- mutant displayed a seemingly biphasic behaviour for the number, intensity and eccentricity of RCs.

## Discussion

The formation of RCs and the association of viral and cellular molecules to these structures have been studied using a variety of experimental strategies that have relied mostly on fluorescence or electron microscopy^24^,^25^,^26^,^27^,^28^,^29^,^30^,^31^,^32^,^33^. Because the viral DBP associates with ssDNA and participates directly in viral DNA replication, many studies have used this protein as a bona fide marker of RCs. The data produced by such studies indicate that RCs assemble in association with the viral DNA, where transcription and post-transcriptional processing of viral genes progressively results in the recruitment to these sites of viral and cellular proteins that direct the various steps of regulated gene expression^27^. As the late phase of viral replication progresses RCs display changes in their size, number and shape. And although it is clear that the dynamics of their formation parallel not only progression of the viral replication cycle, but also the inhibition of cellular defenses, quantitative studies of the dynamic behaviour of RCs have not been conducted. The adjustment of the viral RCs using ellipses presented in this work allowed us to obtain for the first time relevant quantitative data, such as the area, perimeter and form of RCs within the nuclei of infected cells, enabling statistical analysis of these parameters at various time-points of the adenoviral replication cycle.

During the earliest times post-infection, the initial localization of DBP was detected in a few small nuclear foci (at 12 hpi), followed by a progressive increase in their number and size. At time-points post-infection that are known to correspond to the initiation of viral DNA synthesis and the resulting transition to the late phase of the viral replication cycle (between 20 and 24 hpi), larger spherical or doughnut-shaped structures were prominent. Both of these structures increased in number at the later time-point (32–36 hpi). At very late time (36 hpi) some RCs coalesce, resulting in decreased precision of the algorithm. Therefore, except for the latest time-points, when the likelihood of RCs fusion becomes higher, and the adjustment using ellipses can be used for an initial approximation with other strategies, like Level Set, or other dynamic instead of global thresholding methods, the detection of RCs throughout viral replication by the algorithm is in agreement with the qualitative description that has been reported previously by various studies^24^,^27^,^34^,^35^.

In addition to the qualitative description of RCs formation the algorithm allowed us to obtain quantitative measurements that had hitherto not been performed. The statistical analysis of RCs from Ad5 wt compared with the E1B- mutant showed that the absence of the E1B protein is clearly associated with a previously unidentified lower effciency of RCs formation and altered morphological features of RCs at different time-points of viral replication (Fig. 8). The E1B protein is known to participate in both the selective and efficient expression of viral late genes^36^,^37^,^38^,^39^,^40^ and in the inhibition of cellular factors that participate in the anti-viral cellular response^41^. The association of E1B with these structures correlates also with efficient viral DNA synthesis^37^,^42^,^43^,^44^,^45^,^46^. The accumulated evidence indicates that E1B is a multifunctional protein that participates in various processes of viral replication, and that its timely synthesis and localization are necessary for the protein to function as a transcriptional repressor of interferon-inducible genes^47^,^48^, and presumably formation of RCs. The latter, however, has not been demonstrated and the data obtained here allowed us to quantitatively determine for the first time that the E1B impacts each of the parameters used to measure RCs formation. In the absence of E1B, formation of RCs between 12 and 16 hpi displayed similar characteristics as Ad wt, however a clear decrease in their number, area, intensity and eccentricity (Fig. 8), as well as a marked difference in the hierarchical clustering of the 12 hpi time-point (Fig. 9) suggest this early protein may be required for the initial steps of RCs formation. Moreover, the large differences in the range and distribution of the average fluorescence intensity, which indicate variations in the level of DBP that is associated to different RCs, suggest that in the absence of E1B fewer of the DBP-containing small foci that form early or increase in size and give rise to the larger doughnut-shaped structures. These results were not anticipated and suggest that E1B may impact the association of DBP to RCs; experiments are currently in progress to assess this possibility.

The defects displayed by the E1B- mutant virus were not limited to the early time-points. In the absence of this protein the violin plots (Fig. 8) displayed a sine behaviour with the time post-infection, where similar times were observed for the maximum number and intensity of RCs (32 hpi), which paralleled the times for the maximum value for eccentricity (especially at 28 hpi). Interestingly, while hierarchically clustering the variables used to analyze RCs formation dynamics in Ad5 wt-infected cells showed that, as expected, any two time-points that are chronologically closer are more similar to each other (between 16 and 28 hpi), and describe a continuous progression of increasing size, number and intensity of RCs as viral replication progresses (Fig. 9), the area and intensity of RCs reach a maximum value at a time that corresponds to the peak of viral DNA synthesis (28 hpi). In stark contrast, the E1B- mutant displayed alterations in the hierarchical clustering of variables. Notably, the 20 to 24 hpi time-points, which correspond to the initiation of viral DNA synthesis, were no longer grouped together, suggesting that the absence of E1B results in a defect in the adequate formation of RCs that correlates and may be directly linked to the initiation of viral DNA replication.

In summary, in this work the development of a simple and efficient approach to detect and quantitatively measure viral RCs is presented. The proposed algorithm is automatic, non iterative and can detect multiple ellipses of any size and eccentricity. It is deterministic, has a linear algorithmic complexity, and is robust against noisy datasets, even with partial occlusion. This algorithm does not depend on the size or resolution of the images and is invariant to affine transformations. Quantitative statistic analyses of RCs could detect some defects in the formation and maturation of RCs in cells infected with the E1B- mutant virus, indicating that the algorithm represents a simple and powerful tool with predictive capabilities that can be used for the detailed study of RCs formed in cells infected with adenovirus as well as other viruses that induce formation of RCs.

## Methods

### Cells and viruses

Primary human foreskin fibroblasts (HFFs) were maintained in monolayer cultures in Dulbecco’s modified Eagle’s medium supplemented with 10% (vol/vol) fetal calf serum (Gibco-Invitrogen Corp.) for no more than 14 passages. HFFs were infected with Ad5, wt or with an E1B- mutant at 30 PFU/cell, as described previously^39^. The Ad5 H5pg4100 and Ad5 H5pm4149 viruses^49^,^50^ were propagated in monolayers of HEK-293 cells. Viruses were titered as fluorescent forming units (FFU) on HEK-293 cells as described previously^49^.

### Antibodies

The primary antibodies used for immunofluorescence assays were rabbit polyclonal Ab specific for Ad5 DBP (a kind gift from Thomas Dobner, Heinrich-Pette Leibniz Institute, Hamburg, Germany). The secondary antibodies used were, either anti rabbit Alexa Fluor 350 or anti rabbit Alexa fluor 488 (Invitrogen). DAPI was used to stain DNA.

### Fluorescence microscopy

For immunofluorescence, HFF cells grown on coverslips to approximately 90% confluence were mock-infected or infected with Ad5 wt or E1B- mutant. Cells were processed for immunofluorescence as described previously^39^. After application of specific primary antibodies, cells were incubated with secondary antibodies. The cover-slips were mounted on glass slides in PBS/10% glycerol and samples were examined using a Zeiss Axiovert 200 M inverted microscope with a 63*x*/1.4- numerical-aperture oil-immersion objective lens with an Axiocam MRM and Axiovision 3.1 software (Carl Zeiss, Inc.). For each condition 60 2*D* images were collected. The final images were created with ImageJ *v*1.48*o* and were later analyzed in Matlab *R*2011*b*.

### Implementation and details about the proposed algorithm

The objective of the thresholding algorithm is to separate the background from the foreground. Because fluorescence images can have different levels of intensity in the background and it is possible that some columns will not have any information for the RCs, we compute the minimum of the mean intensity of all columns to estimate the average intensity of the background. The standard deviation for the columns was taken into account to estimate the maximum variation of the intensity.

The third step of our algorithm uses the DLSFE approach to adjust the RCs using ellipses, details of this method are presented below:

#### Direct Least Square Fitting Ellipses (DLSFE)

The equation of a conic is given by^11^:


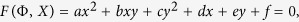


where Φ = [*a*, *b*, *c*, *d*, *e*, *f* ]^*t*^ (parameters) and *X* = [*x*^2^, *xy*, *y*^2^, *x*, *y*, 1]^*t*^ (data points in 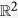). Given *n* points 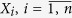 the adjustment to the general form of a conic can be seen like the next minimization problem:


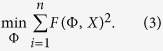


The restriction *b*^2^ − 4*ac* < 0 guarantees that the conic *F*(Φ, *X*) is an ellipse, and since *F*(Φ, *X*) and *αF*(Φ, *X*) is the same conic for all scalar *α* ≠ 0, it is possible to rescale the parameters and consider the next restriction:


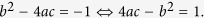


The problem (3) can be expressed as:


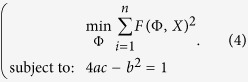


The matrix form of the equation (4) is given by:


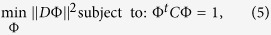


where


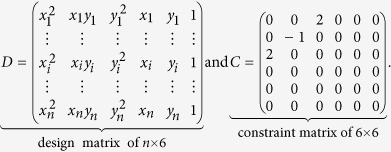


The following system is obtained using Lagrange multipliers and differentiating in problem (5):


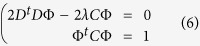


which can be expressed as the equivalent system,


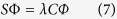



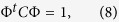


where *S* is the dispersion matrix:


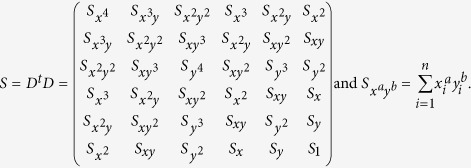


The representation (7) is solved using generalized eigenvectors. In this case the solution is composed of 6 real eigenvalues because the matrices *C* and *D* are symmetrical. If (*λ*_*i*_, *u*_*i*_) (*u*_*i*_ is an eigenvector associated to an eigenvalue *λ*_*i*_) solve (7), then for all *μ*, the pair (*λ*_*i*_, *μu*_*i*_) is the solution to (7). Taking into account the equation (8):


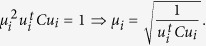


The solutions of the system (6) are of the form 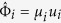. Since there are six solutions, the problem (5) is solved considering the solution associated to the less positive eigenvalue *λ*_*i*_.

All methods have been coded and run in Matlab R2011b (7.13.0.564), under Linux-Ubuntu 14.04, with a processor Intel(R) Core(TM) i5-2450M CPU @ 2.50 GHz and 16 GB of RAM.

### Generation of synthetic images

The steps to generate the synthetic images are the following^17^:

1. Create an image *A* containing ellipses of different sizes, eccentricities and positions according to desired characteristics.
2. Convolute *A* using the point spread function (PSF). For this step we use the plug-in “Diffraction PSF-3D” of ImageJ with the following parameters: Index of refraction of the media *n* = 1.33, Numerical Aperture *NA* = *n* sin (*θ*) = 1.3, Wavelength *γ* = 510 *nm*, Image Pixel Spacing *px* = 100 *nm*, Width (pixels) = 512, Height (pixels) = 512. Rayleigh resolution *r* = 0.61*λ*/*NA* = 240 *nm*. For more details of this plug-in see^51^.
3. Add Gaussian noise in order to consider auto-fluorescence and Poisson noise for the purpose of considering electronic noise. Finally, amplification in an EM-CCD detector has been included^17^. The description of the method to compute the SNR has been included as Supplementary Information.

### Generation of partial occlusion in synthetic images

Each synthetic image has *n* viral RCs simulated through ellipses, whose implicit equation parameters are known. In order to simulate the partial occlusion of the RCs the representation in polar coordinates of the ellipse is considered:


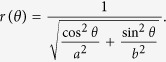


**Figure 10:**
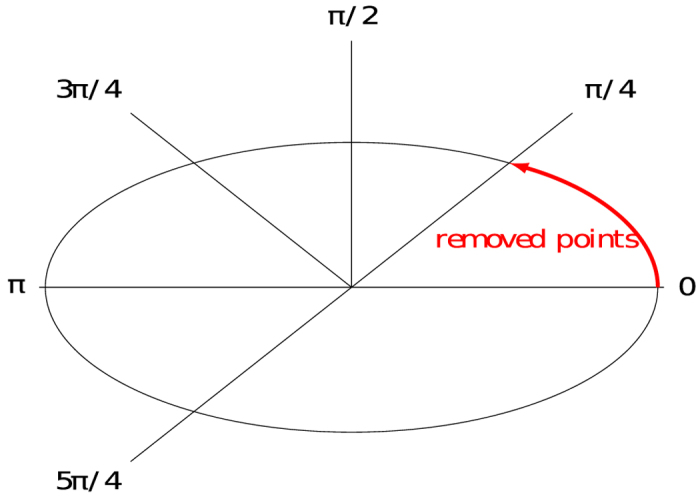
Partial occlusion simulation.

Varying *θ* in a specific interval, points are generated only in the arc determined by this interval, which can be considered as a simulated effect of partial occlusion. Figure 10 shows an example of the ellipse arc generated for 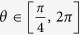, the information enclosed into the angular interval 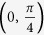 is eliminated as a consequence of the partial occlusion.

The percentage of points conserved with respect to the total, based on the partial occlusion angle is given by:


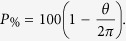


In the case of Fig. 10, 87.5% of total data is conserved.

### Validation of the algorithm

The validation of the algorithm was conducted using measures like the Jaccard index, the Recall and the Precision. Hereinafter each ellipse is represented as a vector of four components *E* = (*a*, *b*, *cx*, *cy*), where the elements of this vector are the coefficients of the implicit form of the ellipse, that is:


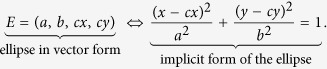


Let 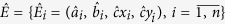 the set of *n* ellipses that simulate the RCs in the image *I*, and 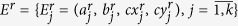 the set of *k* ellipses obtained as a result of the segmentation of the image *I*. The sets 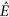 and *E*^*r*^ can be compared following the next rules of classification:

1. True Positive (TP): 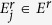 is a true positive (good adjust) if:


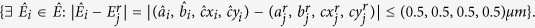


That is, that the absolute error in each coefficient is less than 0.5 *μm*.

2. False Positive (FP): 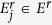 is a false positive (bad adjust) if:


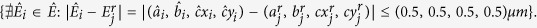


3. False Negative (FN): 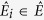 is a false negative if:


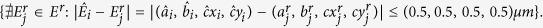


A true positive is a detected ellipse that is similar enough to one of the synthetic ellipses. A false positive is a detected ellipse that is not similar enough to any of the synthetic ellipses and a false negative is a synthetic ellipse that was not detected.

The Jaccard index^52^ is defined as:


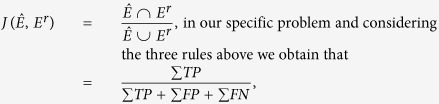


where 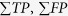 and 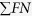 indicate the total number of true positive, false positive and false negative respectively.

The Recall^53^ in the context of our application can be referred as the true positive rate or sensitivity, and it is defined as:


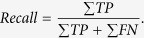


On the other hand, the Precision^53^ is also referred to as positive predictive value, and it is defined as:


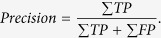


The 0.5 *μm* threshold was chosen based on the resolution limit of optical microscopes, which in agreement with Rayleigh’s criteria, is *r* = (0.61*λ*)/*NA*, with *λ* being the emission wavelength of the fluorophore and *NA* the numerical aperture of the objective. For fluorophores that emit in the visible spectrum the optical resolution of a common epifluorescence microscope is approximately 0.25 − 0.3 *μm* (along the *xy* imaging plane). For a single emitter, *r* approximates the radius of the zero order Airy ring. For the sake of simplicity, we define a 0.5 *μm* threshold as an approximation to the diameter of the zero order Airy ring, because in diffraction limited images it does not make sense to segment replication compartments smaller than 2*r*. Moreover, transmission electron microscopy (TEM) has shown that replication centers measure between 0.5 and 2 *μm* in diameter^54^.

### Statistical analyses

All statistical tests were performed using R version 3.2.4 (2016-03-16) software.

## Additional Information

**How to cite this article**: Garcés, Y. *et al*. Automatic detection and measurement of viral replication compartments by ellipse adjustment. *Sci. Rep*. **6**, 36505; doi: 10.1038/srep36505 (2016).

**Publisher’s note:** Springer Nature remains neutral with regard to jurisdictional claims in published maps and institutional affiliations.

## Supplementary Material

Supplementary Information
