# Supplementary material for: Automatic detection and measurement of viral replication compartments by ellipse adjustment
Source: Sci Rep. 2016 Nov 7;6:36505. doi: 10.1038/srep36505 (PMC5098162; doi:10.1038/srep36505)
Supplement: Supplementary Information [file srep36505-s1.pdf]

Supplementary Information:

Automatic detection and measurement of viral replication compartments by ellipse adjustment

Yasel Garcés,[1] Adán Guerrero,[3,4] Paloma Hidalgo,[2,3]
Raul Eduardo López,[2,3] Christopher D. Wood,[3,4]
Ramón A. Gonzalez,[2] Juan Manuel Rendón-Mancha[1*]

[1] Centro de Investigación en Ciencias,
Instituto de Investigación en Ciencias Básicas y Aplicadas,
Universidad Autónoma del Estado de Morelos (UAEM),
Cuernavaca, Morelos, México.
[2] Centro de Investigación en Dinámica Celular,
Instituto de Investigación en Ciencias Básicas y Aplicadas,
Universidad Autónoma del Estado de Morelos (UAEM),
Cuernavaca, Morelos, México.
[3] Instituto de Biotecnología,
Universidad Nacional Autónoma de México (UNAM),
Cuernavaca, Morelos, México.
[4] Laboratorio Nacional de Microscopía Avanzada,
Instituto de Biotecnología,
Universidad Nacional Autónoma de México (UNAM),
Cuernavaca, Morelos, México.
[*]To whom correspondence should be addressed; E-mail: rendon@uaem.mx

1 Supplementary figure for the algorithm validation.

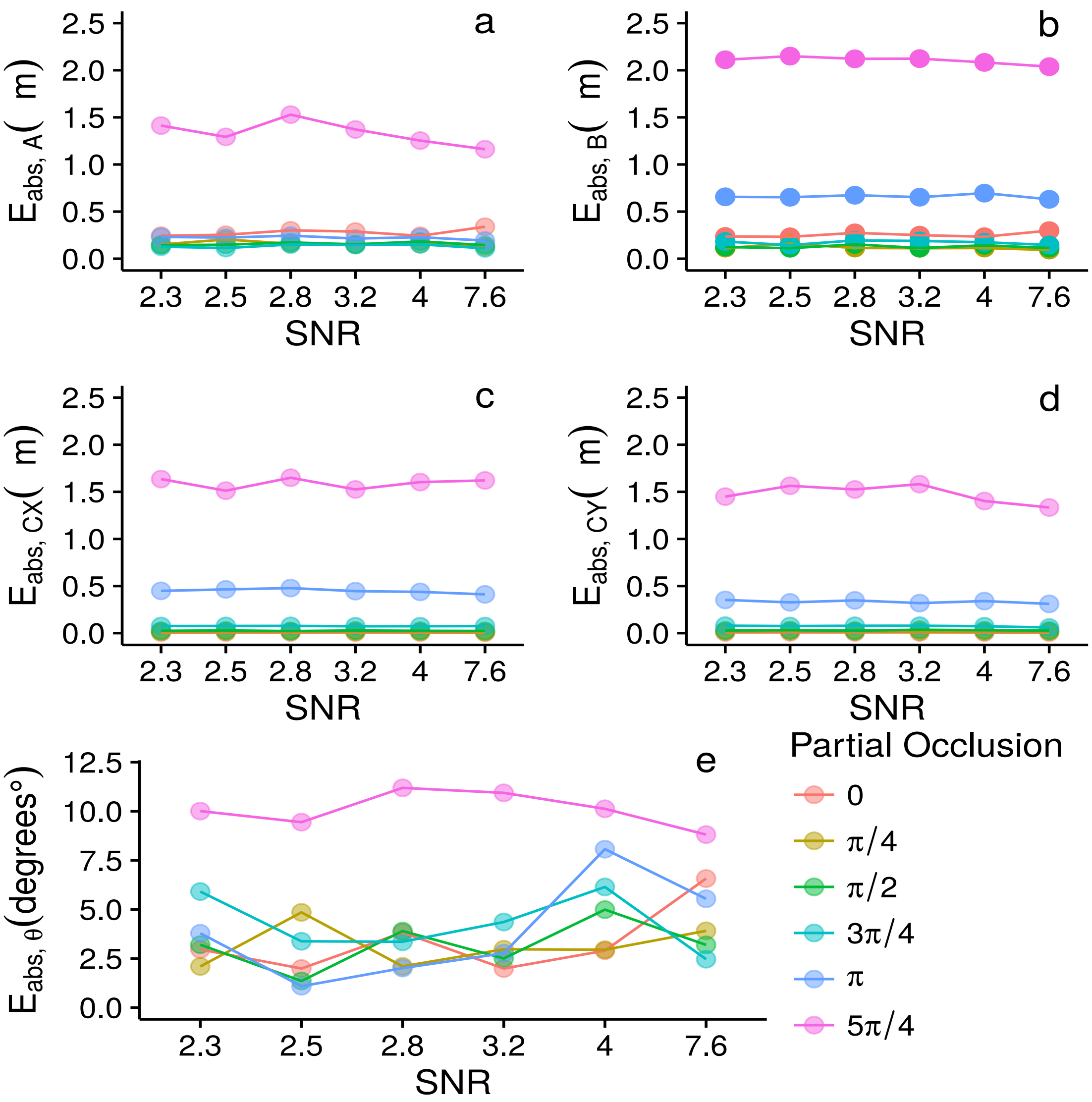

Figure 1: Mean of the absolute error for the coefficients of the ellipse considering different levels of partial occlusion and signal/noise ratios (SNR). In all panels the x-axis is the SNR and each curve represents the results for a specific angle of partial occlusion. The implicit form of an ellipse is given by $(x - CX)^2/A^2 + (y - CY)^2/B^2 = 1$, where (CX, CY) is the center, the coefficients A and B are the major and minor semi-axis, respectively. Figure 1(a)-1(d), represent the error in μm (y-axis) for the estimation of the coefficients $A,\ B,\ CX$ and CY, respectively. Figure 1(e), shows the error in degrees (y-axis) for the rotation angle of the ellipse.

2 SNR computation

The signal/noise ratios (SNR) were computed according to the definitions of Gonzalez (*1*):

$$SNR = 10\log_{10}\left(\frac{mean(Signal^2)}{mean(Noise^2)}\right), \quad (1)$$

where

$$mean(Signal^2) = \overline{R(x,y)^2} = \frac{\sum_{i=1}^{m}\sum_{j=1}^{n}\left[R(x_i,y_j)\right]^2}{m\times n} \quad (2)$$

$$mean(Noise^2) = \overline{\left[R(x,y)-C(x,y)\right]^2} = \frac{\sum_{i=1}^{m}\sum_{j=1}^{n}\left[R(x_i,y_j)-C(x_i,y_j)\right]^2}{m\times n}. \quad (3)$$

Note that the ground truth image $R(x,y)$ (signal) and the corrupted image $C(x,y)$ (contains signal and noise) are size $m\times n$. Then, replacing (2) and (3) in the equation (1) we obtain:

$$SNR = 10\log_{10}\left[\frac{\sum_{i=1}^{m}\sum_{j=1}^{n}\left[R(x_i,y_j)\right]^2}{\sum_{i=1}^{m}\sum_{j=1}^{n}\left[R(x_i,y_j)-C(x_i,y_j)\right]^2}\right]. \quad (4)$$

3 Source Code

Matlab codes to process the images are available in Github (Source Codes). The terms and conditions for the use and distribution of this code are explained in the README file.

References and Notes

1. R. Gonzalez and R. Woods. *Digital Image Processing*. Prentice Hall, third edition edition, 2008.

2. R. Halir and J. Flusser. Numerically stable direct least-squares fitting of ellipses. In *Sixth International Conference of Computer Graphics and Visualization*, 1998.
